# Association Between High Serum Anion Gap and All-Cause Mortality in Non-Traumatic Subarachnoid Hemorrhage: A Retrospective Analysis of the MIMIC-IV Database

**DOI:** 10.3389/fneur.2022.922099

**Published:** 2022-07-12

**Authors:** Changli Zhong, Min Ye, Liyi Hu, Jiuling Liu

**Affiliations:** ^1^Department of Clinical Laboratory, The First People's Hospital of Chongqing Liang Jiang New Area, Chongqing, China; ^2^Department of Neurology, Nanjing BenQ Medical Center, The Affiliated BenQ Hospital of Nanjing Medical University, Nanjing, China

**Keywords:** non-traumatic subarachnoid hemorrhage, serum anion gap, mortality, MIMIC-IV database, critically ill

## Abstract

**Background:**

High serum anion gap (AG) on admission is often correlated with poor outcomes in critically ill patients; however, data in patients with non-traumatic subarachnoid hemorrhage (SAH) are lacking. Herein, we aimed to identify the association between serum AG and all-cause mortality in patients with non-traumatic SAH.

**Methods:**

A retrospective analysis of data from the Medical Information Mart for Intensive Care (MIMIC-IV) database was performed on critically ill patients with non-traumatic SAH. Serum AG was collected on Intensive Care Unit (ICU) admission, and ICU and hospital all-cause mortality were analyzed. The multivariate Cox proportional hazard regression model and Kaplan-Meier survival curve analysis were used to analyze the correlation of serum AG with ICU and hospital all-cause mortality. Furthermore, interaction and subgroup analyses were evaluated for the consistency of these correlations.

**Results:**

A total of 893 patients with non-traumatic SAH were included in this study. The all-cause mortality in ICU and hospital were 14.8% (132/893), and 18.9% (169/893), respectively. Multivariate analysis after adjusting for potential confounders indicated that high serum AG levels (≥16 mmol/L) were associated with increased risk of ICU and hospital all-cause mortality as compared to that with low serum AG levels (<16mmol/L), (hazards ratio (HR): 2.31 [95% CI: 1.58–3.38]) and HR: 1.91 [95% CI: 1.36–2.67)], respectively). Similarly, the Kaplan–Meier (K–M) survival curve also showed that patients with high serum AG levels presented with a lower survival rate. Stratified analyses further showed that depending on the variable testes, an association between higher serum AG levels and hospital all-cause mortality in different subgroups was observed.

**Conclusion:**

Among patients with non-traumatic SAH, high serum AG level at ICU admission was associated with increased ICU and hospital all-cause mortality.

## Introduction

Non-traumatic subarachnoid hemorrhage (SAH) is a potentially devastating disease caused primarily by ruptured intracranial aneurysms, which account for 2–7% of all strokes (1). However, the disease-specific burden of non-traumatic SAH is unusually heavy and may be underestimated. Half of the patients with non-traumatic SAH are reported to be younger than 60 years, of which one-third are reported to expire before arrival at the hospital, while others required Intensive Care Unit (ICU) treatment (1, 2). Even with optimal management in the ICU, non-traumatic SAH still has high in-hospital mortality rates (3). Epidemiological investigations have shown high rates of non-traumatic SAH and in-hospital mortality rates of up to 40% (1). Given the life-threatening risk of non-traumatic SAH, non-invasive and inexpensive tests are needed to identify those at greater risk of death and prevent mortalities.

Acid-base imbalance, which has been mainly reported and investigated in critically ill patients, has been correlated with poor outcomes, especially in cases of persistent acid-base imbalance (4). The serum anion gap (AG) is a useful biochemical indicator for evaluating acid-base balance in clinical practice and is easily obtained (5) using the formula: AG = [Na^+^ (mmol/L) + K^+^ (mmol/L)] – [Cl^−^(mmol/L) + ${\text{HCO}}_{3}^{-}$ (mmol/L)]. Our stress response during and after the onset of non-traumatic SAH is complicated and leads to disturbance in the inner environment. Many studies have demonstrated that serum AG was strongly related to mortality in critically ill patients, specifically in individuals with congestive heart failure (6), cardiogenic shock (7), ischemic stroke (8, 9), acute kidney injury (10), acute pancreatitis (11), acute myocardial infarction (12) and aortic aneurysm (13). Although the effect of serum AG on mortality following ischemic stroke has been well described (8, 9); however, data in patients with non-traumatic SAH are lacking.

Therefore, the purpose of this study was to clarify the correlation between serum AG levels and mortality in patients with non-traumatic SAH in the ICU.

## Methods

### Data Source

We performed a retrospective cohort study using the Medical Information Mart for Intensive Care (MIMIC-IV) (version 1) (14), which is a large publicly accessed database.

The use of this database has been approved by the Massachusetts Institute of Technology and the Institutional Review Board of Beth Israel Deaconess Medical Center (BIDMC, Boston, MA, USA). One of the authors, Changli Zhong, accomplished the National Institutes of Health's web-based course “Protecting Human Research Participants” (Record ID: 39099161) and was approved to access the database to extract data. To protect patient privacy, all data were de-identified. Thus, informed consent was waived by the ethical committee of the Beth Israel Deaconess Medical Center. This study is described in conformity to the STrengthening the Reporting of OBservational studies in Epidemiology (STROBE) statement and was managed to conform to the tenets of the Declaration of Helsinki.

### Study Population and Variable Extraction

**Figure 1:**
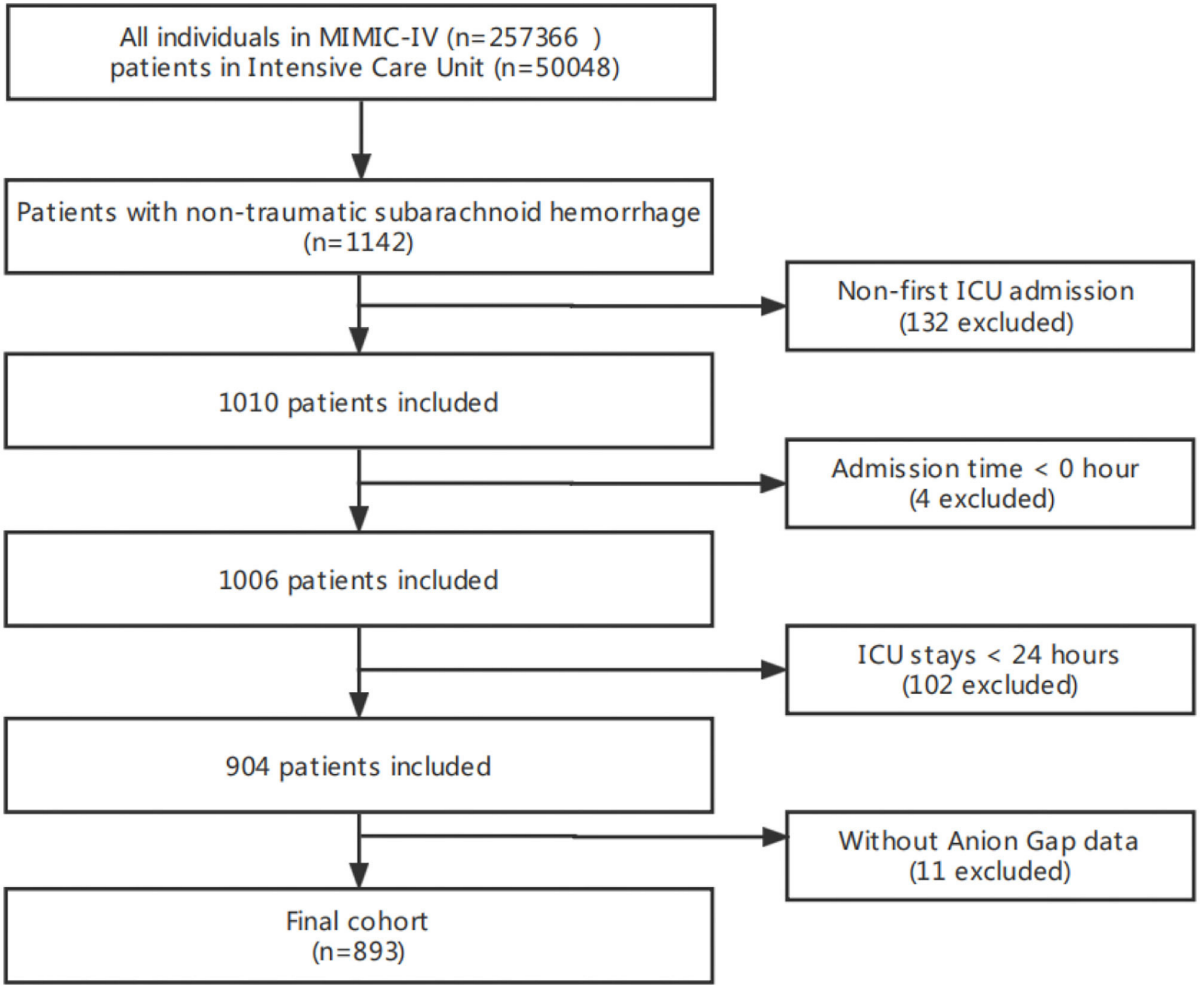
A flowchart of study patients.

The total number of patients in the MIMIC-IV included 257,366 individuals from 2008 to 2019, of which 50,048 were admitted to the ICU. Among them, 1,142 patients with non-traumatic SAH were selected based on the record of ICD-9 code 430, and ICD-10 codes I60, I600 to I6012, I6000 to I6002, I6020 to I6022, I6030 to I6032, and I6050 to I6052. Patients >18 years were initially enrolled in this study, and only data for the first ICU stay were collected for patients. Meanwhile, patients without serum AG level after ICU admission who stayed in the ICU for <24 h and had a survival time of <0 h, were all excluded. Thus, only 893 patients were included in this study (Figure 1).

The first test serum AG value after ICU admission was extracted as the interest variable and the major exposure factor in this study. All variables in this study were extracted from the MIMIC-IV database using Structured Query Language (SQL) with PostgreSQL. Demographic variables, including age, sex, and ethnicity, were obtained. Clinical severity on admission was examined using the Glasgow Coma Score (GCS) and Simplified Acute Physiology Score II (SAPS II). Vital signs in this study also included, such as systolic blood pressure (SBP), diastolic blood pressure (DBP), mean blood pressure (MBP), temperature, heart rate, respiratory rate (RR), and percutaneous oxygen saturation (SpO_2_). Comorbidities, including hypertension, diabetes, congestive heart failure, chronic pulmonary disease, sepsis, renal failure, liver diseases, and malignancy, were also included for analysis based on the recorded ICD-9 and ICD-10 codes from the database. Laboratory variables, including white blood cell (WBC) count, platelet count, hemoglobin, glucose, sodium, potassium, chloride, creatinine, blood urea nitrogen (BUN), and bicarbonate, were obtained within the first test after ICU admission. For the missing dataset in the data, we used the predicted mean matching method to impute the missing values. The details of the missing value are shown in Table 1.

**Table 1:** Details of missing values.

**Variables**	**The number of**	**The percent of**
	**missing values**	**missing values (%)**
Respiratory rate	2	0.2
Bicarbonate	6	0.7
BUN	1	0.7
Creatinine	1	0.7
Chloride	6	0.7
Hemoglobin	6	0.7
Platelet	1	0.1
WBC	1	0.1

*WBC, white blood cell; BUN, blood urea nitrogen*.

### Outcomes

The primary endpoint was hospital all-cause mortality, and ICU all-cause mortality was regarded as the secondary endpoint, which was defined by patient survival status at the time of hospital discharge.

### Statistical Analysis

Continuous variables were described as means ± standard deviation (SD) or as median interquartile ranges (IQR), and categorical variables were described as percentages. We used Fisher's exact and Chi-square tests or the Kruskal-Wallis test to examine the statistical differences between two groups—the low serum AG (AG <16 mmol/L) and high serum AG (AG ≥ 16 mmol/L) groups. Restricted cubic spline analysis was used to describe the non-linear association between serum AG and ICU and hospital all-cause mortality with non-traumatic SAH subjects. We then used multivariate Cox proportional hazard models to evaluate the relationship of serum AG level with ICU and hospital mortality. Baseline variables that were considered clinically relevant or had a change in effect estimate of >10% were chosen as confounders. In Model I, the covariates were adjusted for age, sex, and ethnicity, whereas in Model II, SBP, DBP, RR, heart rate, GCS, diabetes, sepsis, renal failure, chronic pulmonary disease, vasopressor, embolization of aneurysm, clipping of aneurysm, WBC, hemoglobin, platelet, glucose, BUN, and creatinine were also included in addition to the covariates of Model I. The Kaplan–Meier (K–M) curve was also utilized to visualize these relationships. Furthermore, interactions and stratified analyses were conducted using hypertension, diabetes, congestive heart failure, chronic pulmonary disease, renal failure, sepsis, GCS (≥ 8 and <8), and SAPS II (≥ 45 and <45), as previously described. For the missing data, we used the predicted mean matching method to fill in the missing values (15). All results were expressed as hazard ratios (HR) with a 95% CI, and a *p-*value of <0.05 was considered significant. All analyses were performed using the statistical software packages R 3.3.2 (http://www.R-project.org, The R Foundation) and Free Statistics software version 1.4 (Beijing, China).

## Results

### Baseline Characteristics of Subjects

In total, 893 of the 1,142 non-traumatic SAH patients who received ICU treatment were included in this study (Figure 1). Among them, there were 402 males and 491 females, and the mean age was 61.2 ± 14.9. The distribution of the baseline population characteristics according to serum AG levels in tertiles is described in Table 2. The patients with high serum AG levels (≥16 mmol/L) were found to have a lower GCS score, a higher SAPS II score and mortality, received more vasopressor treatment, and had more commodities, such as diabetes, sepsis, and chronic pulmonary disease.

**Table 2:** The clinical characteristics of critically ill patients with non-traumatic SAH.

**Characteristics**	**Serum Anion Gap (mmol/L)**				***p*-value**
	**Total**	**Tertile1(<13)**	**Tertile2(≥13, <16)**	**Tertile3(≥16)**	
	**(*n =* 893)**	**(*n =* 212)**	**(*n =* 372)**	**(*n =* 309)**	
**Age, years**	61.2 ± 14.9	62.1 ± 15.0	60.1 ± 14.6	62.0 ± 15.2	0.172
**Sex**, ***n*** **(%)**					0.791
Male	402 (45.0)	99 (46.7)	163 (43.8)	140 (45.3)	
Female	491 (55.0)	113 (53.3)	209 (56.2)	169 (54.7)	
**Ethnicity**, ***n*** **(%)**					0.005
White	541 (60.6)	138 (65.1)	238 (64)	165 (53.4)	
Asian	31 (3.5)	5 (2.4)	17 (4.6)	9 (2.9)	
Black	69 (7.7)	17 (8)	30 (8.1)	22 (7.1)	
other	252 (28.2)	52 (24.5)	87 (23.4)	113 (36.6)	
SBP, mmHg	125.1 ± 13.0	124.1 ± 12.8	125.4 ± 13.1	125.5 ± 13.1	0.445
DBP, mmHg	64.2 ± 9.0	62.6 ± 7.9	64.7 ± 9.0	64.7 ± 9.5	0.011
MBP, mmHg	82.4 ± 8.7	81.2 ± 7.9	82.8 ± 9.1	82.7 ± 8.8	0.078
HR, beats/min	78.6 ± 13.1	76.4 ± 11.9	77.1 ± 12.6	82.0 ± 13.9	<0.001
RR, beats/min	18.2 ± 3.3	17.6 ± 2.6	17.7 ± 3.2	19.3 ± 3.6	<0.001
Temperature, °C	37.0 ± 0.5	37.0 ± 0.4	37.0 ± 0.4	37.0 ± 0.7	0.788
SpO2, (IQR)	97.6 (96.1, 98.9)	97.7 (96.4, 99.0)	97.6 (96.0, 98.8)	97.6 (96.0, 98.9)	0.433
ICU length of stay, days	9.2 ± 8.5	8.4 ± 8.3	9.8 ± 9.1	9.0 ± 8.0	0.123
Hospital length of stay, days	14.7 ± 12.4	13.8 ± 11.6	15.6 ± 13.0	14.1 ± 12.2	0.149
ICU mortality, *n* (%)	132 (14.8)	25 (11.8)	25 (6.7)	82 (26.5)	<0.001
Hospital mortality, *n* (%)	169 (18.9)	30 (14.2)	44 (11.8)	95 (30.7)	<0.001
**Surgery**, ***n*** **(%)**					0.229
Embolization of aneurysm	36 (4.0)	6 (2.8)	20 (5.4)	10 (3.2)	
Clipping of aneurysm	786 (88.0)	184 (86.8)	323 (86.8)	279 (90.3)	
**Vasopressor**, ***n*** **(%)**	38 (4.3)	5 (2.4)	10 (2.7)	23 (7.4)	0.003
**Scoring systems**
GCS	10.6 ± 4.1	11.2 ± 3.8	10.8 ± 4.0	9.8 ± 4.3	<0.001
SAPS II	32.4 ± 13.0	30.8 ± 11.6	30.4 ± 11.6	35.9 ± 14.8	<0.001
**Comorbidities**, ***n*** **(%)**
Hypertention	446 (49.9)	96 (45.3)	187 (50.3)	163 (52.8)	0.243
Diabetes	127 (14.2)	27 (12.7)	39 (10.5)	61 (19.7)	0.002
Congestive heart failure	74 (8.3)	17 (8)	27 (7.3)	30 (9.7)	0.507
Chronic pulmonary disease	128 (14.3)	46 (21.7)	41 (11)	41 (13.3)	0.002
Sepsis	441 (49.4)	95 (44.8)	170 (45.7)	176 (57)	0.004
Renal failure	63 (7.1)	13 (6.1)	22 (5.9)	28 (9.1)	0.233
Liver diseases	14 (1.6)	3 (1.4)	5 (1.3)	6 (1.9)	0.802
Malignancy	35 (3.9)	5 (2.4)	15 (4)	15 (4.9)	0.35
**Laboratory tests**
WBC (10^9^/L)	12.6 ± 5.7	11.4 ± 5.7	12.3 ± 5.3	13.9 ± 5.9	<0.001
Hemoglobin (g/dL)	12.8 ± 2.1	12.3 ± 2.2	12.9 ± 2.0	13.0 ± 2.2	<0.001
Platelet (10^9^/L)	227.1 ± 89.8	221.8 ± 82.6	229.9 ± 90.7	227.4 ± 93.4	0.573
Glucose(mmol/L)	150.1 ± 58.3	134.7 ± 36.2	148.1 ± 56.9	163.2 ± 68.7	<0.001
Sodium (mmol/L)	138.9 ± 4.0	139.2 ± 4.0	139.0 ± 3.9	138.5 ± 4.2	0.169
Potassium (mmol/L)	4.1 ± 0.8	4.1 ± 0.8	4.1 ± 0.8	4.1 ± 0.8	0.857
Chloride (mmol/L)	103.3 ± 4.8	104.6 ± 4.7	103.5 ± 4.5	102.1 ± 5.0	<0.001
BUN (mg/dL)	17.5 ± 11.7	16.5 ± 8.8	16.4 ± 8.6	19.6 ± 15.7	<0.001
Creatinine (mg/dL)	1.0 ± 0.8	0.8 ± 0.4	0.9 ± 0.3	1.1 ± 1.3	<0.001
Bicarbonate (mmol/L)	22.9 ± 3.7	24.2 ± 3.7	23.3 ± 3.5	21.6 ± 3.4	<0.001

*SAH, subarachnoid hemorrhage; SBP, systolic blood pressure; DBP, diastolic blood pressure; MBP, mean blood pressure; RR, respiratory rate; HR, heart rate; SpO2, percutaneous oxygen saturation; SAPS II, simplified acute physiology score II; GCS, Glasgow Coma Score; Chronic pulmonary disease, chronic obstructive pulmonary disease; WBC, white blood cell; BUN, blood urea nitrogen*.

### Association Between Serum AG and All-Cause Mortality in Non-Traumatic SAH

**Figure 2:**
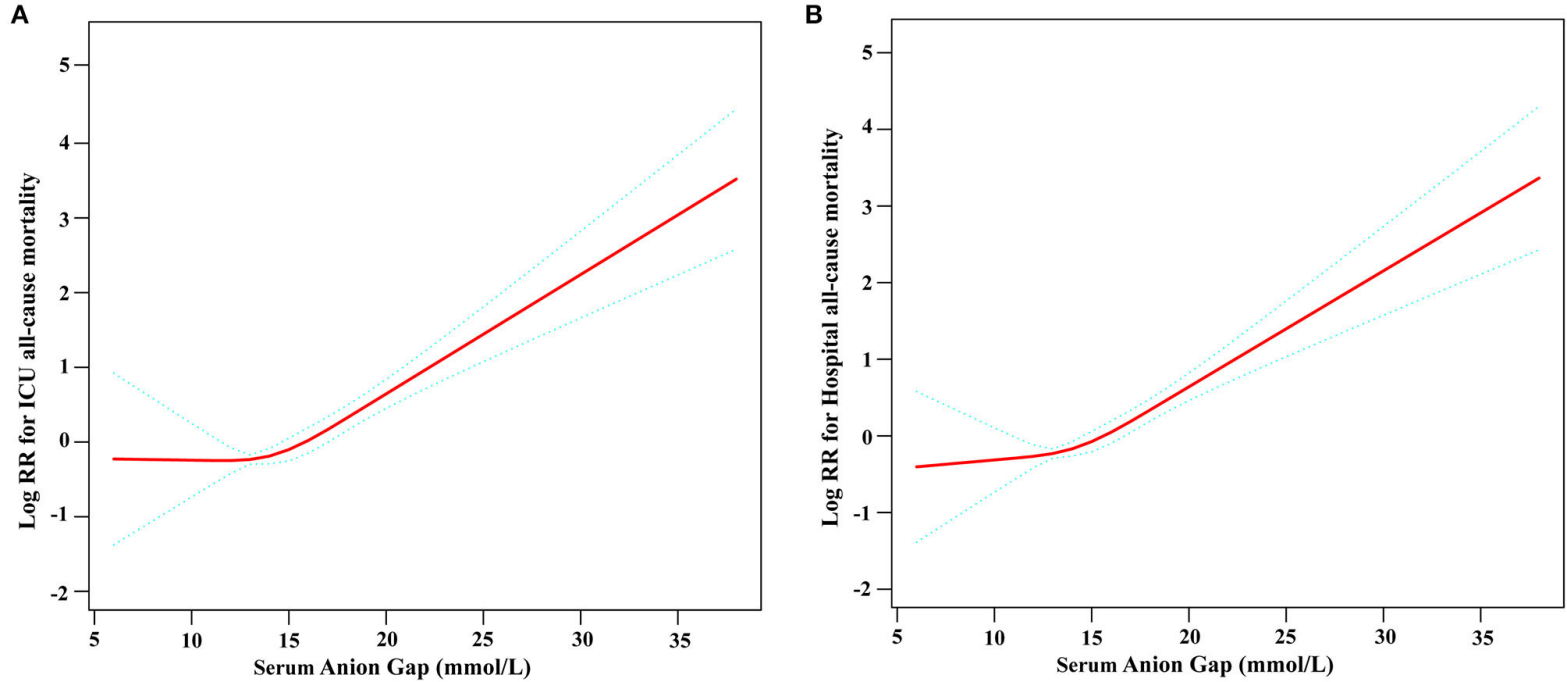
Construction of smooth curve describing the risk of mortality against serum Anion Gap using a restricted cubic spline model. **(A)** ICU all-cause mortality; **(B)** Hospital all-cause mortality. The solid red line represents the smooth curve fit between variables. Blue bands present the 95% confidence interval. Data were adjusted for age, sex, ethnicity, SBP, DBP, respiratory rate, GCS, diabetes, sepsis, renal failure, chronic pulmonary disease, vasopressor, embolization of aneurysm, clipping of aneurysm, WBC, platelet, hemoglobin, glucose, BUN, and creatinine.

Restricted cubic spline analysis revealed a non-linear relationship between serum AG and ICU all-cause mortality in subjects with non-traumatic SAH, which is consistent with hospital all-cause mortality. Serum AG level (<16 mmol/L) was not significantly associated with ICU and hospital all-cause mortality with non-traumatic SAH. There is an increase in ICU and hospital all-cause mortality with non-traumatic SAH as serum AG level (≥16 mmol/L) increases, as shown in Figure 2. Table 3 provides the unadjusted and adjusted analyses for serum AG level and all-cause mortality in patients with non-traumatic SAH using Cox proportional hazards models. When used as a continuous variable, serum AG was associated with an increased risk of ICU (HR: 1.09 [95% CI: 1.04–1.14]) and hospital all-cause mortality (HR: 1.08 [95% CI: 1.03–1.13]). The ICU and hospital all-cause mortality of non-traumatic SAH increased with a per 1-unit increase in serum AG. Serum AG was evaluated as a categorical variable with ICU and hospital all-cause mortality, wherein the lower serum AG level (<16 mmol/L) was considered the reference. In the crude model, high serum AG level was associated with increased risk of ICU (HR: 3.17 [95% CI: 2.23–4.50]) and hospital all-cause mortality (HR: 2.67 [95% CI: 1.96–3.63]), respectively. In Model I, with adjustments for age, gender, ethnicity, and high serum AG level was associated with increased risk of ICU (HR: 2.88 [95% CI: 2.02–4.11]) and hospital all-cause mortality (HR: 2.40 [95% CI: 1.76–3.28]), respectively. Furthermore, Model II, which adjusted for age, sex, ethnicity, SBP, DBP, RR, heart rate, GCS, diabetes, sepsis, renal failure, chronic pulmonary disease, vasopressor, embolization of aneurysm, clipping of aneurysm, WBC, platelet, hemoglobin, glucose, BUN and creatinine, the higher serum AG still remained significantly associated with an increase in ICU (HR: 2.31 [95% CI: 1.58–3.38]) and hospital all-cause mortality (HR: 1.91 [95% CI: 1.36–2.67]) with the low serum AG group as reference. Regarding the sensitivity analysis, serum AG levels were both assessed as a continuous and categorical variable with ICU and hospital all-cause mortality, yielding consistent results.”

**Table 3:** Multivariable cox regression models evaluating the association between serum Anion Gap and ICU and hospital all-cause mortality.

**Variable**	**Crude**		**Model I**		**Model II**	
	**HR(95%CI)**	***p*-value**	**HR(95%CI)**	***p*-value**	**HR(95%CI)**	***p*-value**
**ICU all-cause mortality**
Serum AG <16 mmol/L	1 (Ref)		1 (Ref)		1 (Ref)	
Serum AG ≥16 mmol/L	3.17 (2.23, 4.5)	<0.001	2.88 (2.02,4.11)	<0.001	2.31 (1.58,3.38)	<0.001
Serum AG, 1 mmol/L	1.14 (1.11, 1.18)	<0.001	1.15 (1.11, 1.19)	<0.001	1.09 (1.04, 1.14)	<0.001
**Hospital all-cause mortality**
Serum AG <16 mmol/L	1 (Ref)		1 (Ref)		1 (Ref)	
Serum AG ≥16 mmol/L	2.67 (1.96, 3.63)	<0.001	2.40 (1.76, 3.28)	<0.001	1.91 (1.36,2.67)	<0.001
Serum AG, 1 mmol/L	1.14 (1.10, 1.18)	<0.001	1.14 (1.11, 1.18)	<0.001	1.08 (1.03,1.13)	<0.001

***Crude model**: adjusted for none.*

***Model I**: adjusted for age, sex, and ethnicity.*

***Model II**: adjusted for **Model I**+SBP, DBP, respiratory rate, heart rate, GCS, diabetes, sepsis, renal failure, chronic pulmonary disease, vasopressor, embolization of aneurysm, Clipping of aneurysm, WBC, platelet, hemoglobin, glucose, BUN, creatinine*.

**Figure 3:**
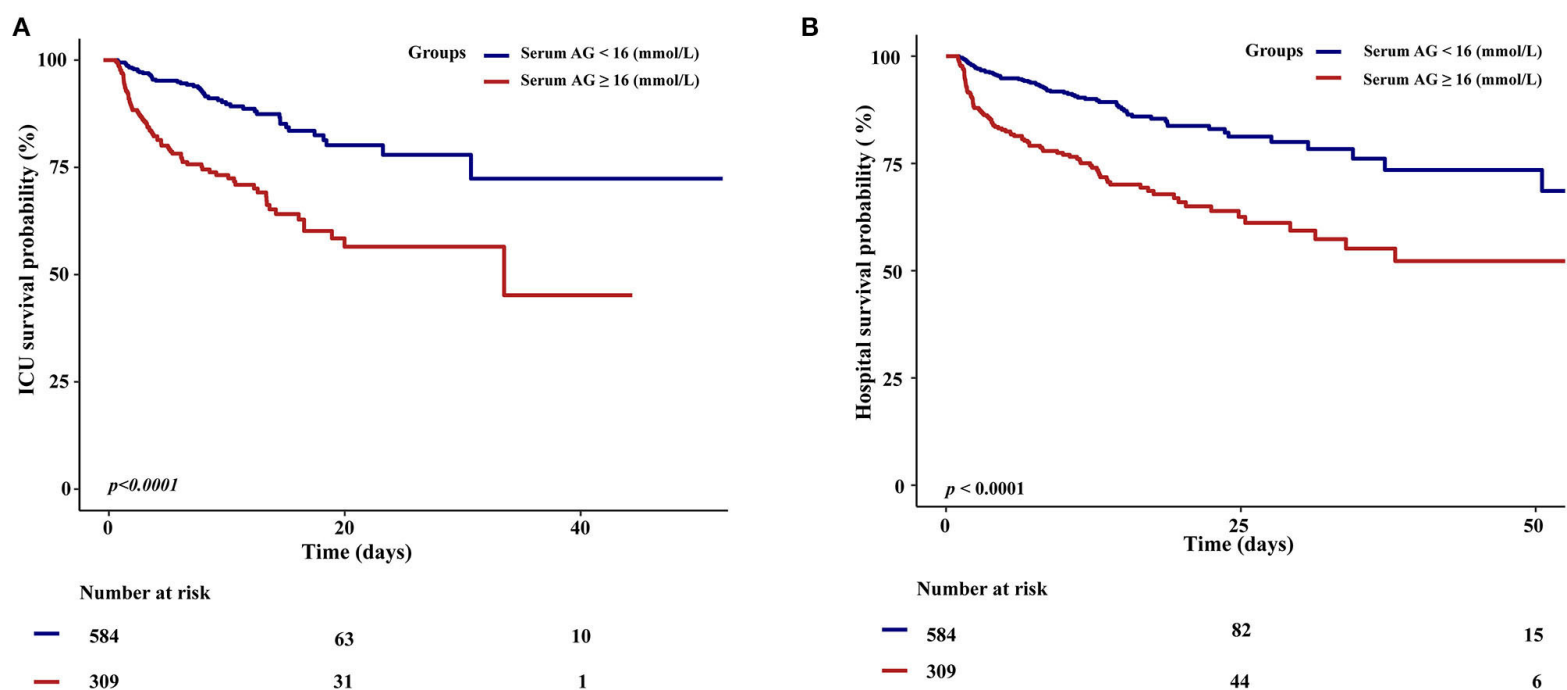
Kaplan–Meier survival curves for critically ill patients with non-traumatic SAH based on serum Anion Gap. **(A)** ICU all-cause mortality; **(B)** Hospital all-cause mortality. *x*-Axis: survival time (days). *y*-Axis: survival probability.

In addition, the KM survival curve demonstrated that patients with high serum AG levels (≥16 mmol/L) on admission were associated with a lower risk of ICU and hospital survival rate (*p* < 0.0001*)* in Figure 3.

### Subgroup Analysis

**Figure 4:**
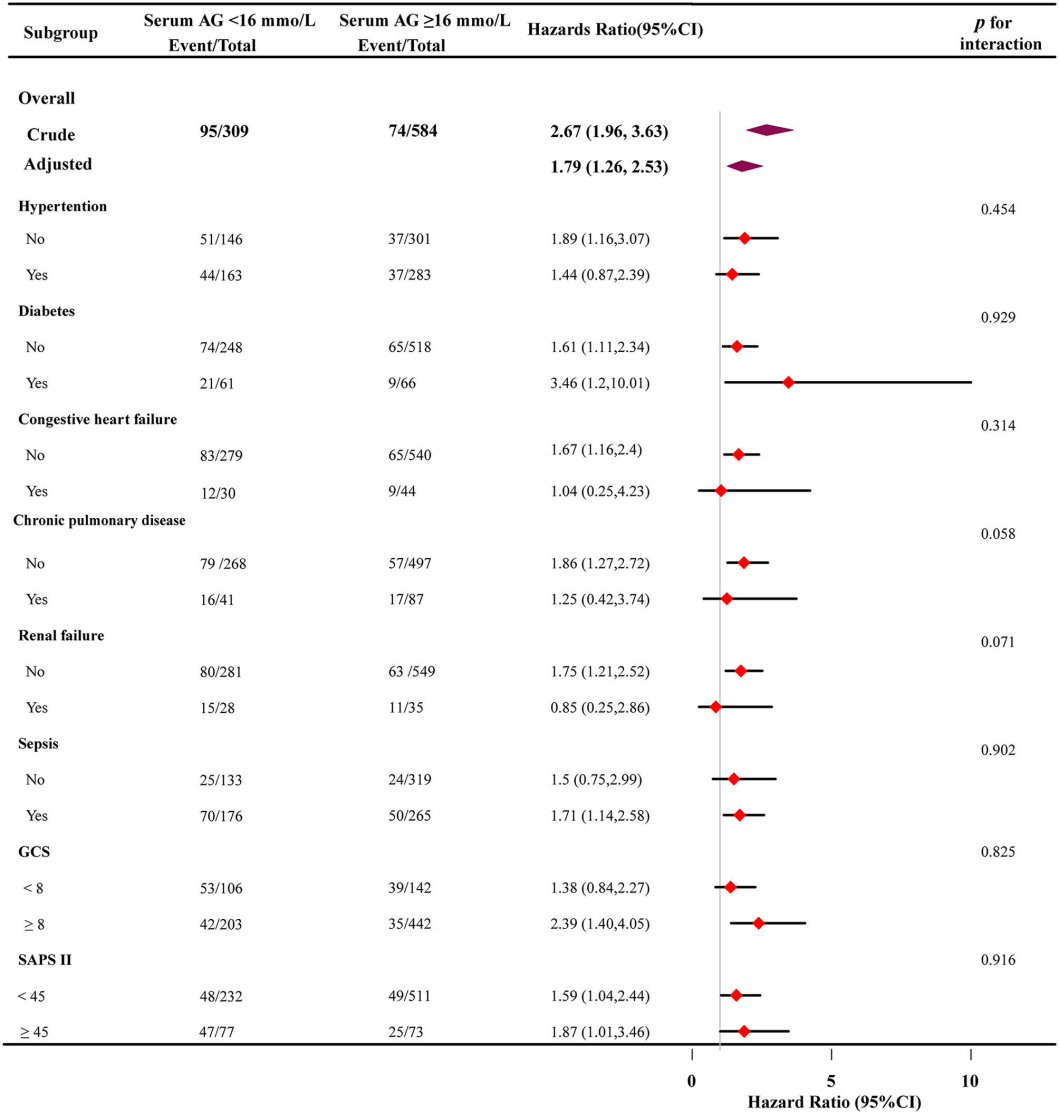
Subgroup analyses of the effect of on hospital all-cause mortality. adjusted for age, sex, and ethnicity, SBP, DBP, respiratory rate, GCS, diabetes, sepsis, renal failure, chronic pulmonary disease, vasopressor, embolization of aneurysm, Clipping of aneurysm, WBC, platelet, hemoglobin, glucose, BUN, creatinine. In each case, the model was not adjusted for the stratification variable.

Subgroup analyses were performed to evaluate the association between high serum AG levels and hospital all-cause mortality (Figure 4). Based on the variables tested, an association between higher serum AG level and hospital all-cause mortality was observed in different subgroups (hypertension, diabetes, congestive heart failure, chronic pulmonary disease, renal failure, sepsis, GCS, and SAPS II) (*p* >0.05 for all).

## Discussion

This study revealed that subjects with high serum AG levels presented with a lower survival rate and shorter survival time, and that high serum AG level on admission was a significant risk for ICU and hospital all-cause mortality in critically ill patients with non-traumatic SAH. Specifically, patients with high serum AG levels (≥16 mmol/L) had a 2.31- and 1.91-fold higher risk of ICU and hospital all-cause mortality, respectively, than those with low serum AG levels. Moreover, we showed that different subgroups had no interactions upon correlating serum AG levels with hospital all-cause mortality.

Serum AG elevation is generally caused by the overproduction of organic acids or the reduced excretion of anions (5). In the clinical setting, serum AG is beneficial due to its simple calculation and because it does not require arterial access, which is why it is routinely determined in all patients admitted to the ICU (13). As such, many studies have explored the relationship between serum AG levels and clinical outcomes of critically ill patients. Recent studies using data from the MIMIC database found that elevated serum AG levels were correlated with an increased risk of all-cause mortality among patients with acute ischemic stroke (8). One study also showed that elevated serum AG levels were significantly associated with mortality in patients with cerebral infarction (9). Similarly, our study results were consistent with previous studies. After adjusting for confounding factors, patients with high serum AG levels ≥ 16 mmol/L) had a 2.31- and 1.91-fold higher risk of ICU and hospital all-cause mortality, respectively, than those with low serum AG levels. Furthermore, a recent systematic review and meta-analysis indicated that serum AG might be a viable tool for assessing the prognosis of critically ill patients, especially in areas with poor medical resources (16). Studies also showed that hypertension, diabetes, congestive heart failure, chronic pulmonary disease, renal failure, and sepsis are common comorbidities in patients with non-traumatic SAH. Although these comorbidities have been associated with poor outcomes, our stratified and subgroup analyses did not change our overall results.

Interestingly, Tang et al. (12) and Cheng et al. (10) reported a U-shaped relationship between serum AG and mortality in critically ill patients with congestive heart failure and acute kidney injury, respectively. Gong et al. (11) also reported this U-shaped relationship between serum AG and ICU mortality in patients with acute pancreatitis. However, these findings may have obscured the relationship considering their insufficient sample size and that the low anion gap was assumed as a laboratory error (17).

It is difficult to clarify the precise mechanism behind the close correlation between serum AG and all-cause mortality in patients with non-traumatic SAH. Nevertheless, we can propose several potential explanations. One explanation is that an intracranial aneurysm rupture often causes a stress response in the body, leading to a disturbance in the internal environment. In addition, the cerebral blood flow (CBF) becomes unstable and brain metabolism is disturbed, leading to changes in ion concentration. Another explanation is that elevated serum AG levels usually manifest as a mild hemodynamic disturbance with inadequate tissue perfusion, which predisposes the patient to cerebral vasospasm (18). A third possibility is that elevated serum AG levels result in increased blood lactate levels, and acidosis due to increased organic acids can aggravate tissue ischemia and hypoxia (19). Once epilepsy is induced by massive bleeding, it can further aggravate ischemia and hypoxia, and tissue hypoxia caused by many factors can cause deterioration of the condition and even lead to death (20). Furthermore, high serum AG levels have been shown to be associated with high levels of inflammatory biomarkers (21). The influence of blood in the subarachnoid space initiates the rapid activation of inflammatory cascades, and the neuroinflammatory response has been found to play an important role in the outcome of patients with non-traumatic SAH (22).

Despite these findings, several limitations of our study should be noted. First, given the retrospective design of the study, data had already been collected. Second, due to the limitations of the MIMIC database, missing information that could have affected the model was not collected, such as albumin, lactate, pH, and Hunt & Hess grades. However, it should be noted that the potential results from these variables would bias toward the null, resulting in an undervaluation of the connection between serum AG levels and all-cause mortality. Third, we were only able to provide the association between serum AG levels and mortality rather than establish a causal relationship. Nevertheless, the relationship between high serum AG levels and all-cause mortality was clearly revealed.

In summary, this retrospective observational study revealed that high serum AG levels were a significant risk for ICU and hospital all-cause mortality in patients with non-traumatic SAH. Further prospective studies with larger sample sizes should be performed to assess the causality between high serum AG levels and ICU and hospital all-cause mortality.

## Data Availability Statement

The data analyzed in this study was obtained from Medical Information Mart for Intensive Care IV (MIMIC-IV, https://mimic-iv.mit.edu), the following licenses/restrictions apply: To access the files, you must be a credentialed user, finish required training and sign the data use agreement for the project. Requests to access these datasets should be directed to PhysioNet, https://physionet.org/, 10.13026/s6n6-xd98.

## Ethics Statement

The studies involving human participants were examined and approved by Beth Israel Deaconess Medical Center. To protect patient privacy, all data were de-identified; therefore, the Ethical Committee of the Beth Israel Deaconess Medical Center waived the requirement for informed consent.

## Author Contributions

JL and CZ designed the study and collected the data. JL, CZ, MY, and LH interpreted the result and interpreted the result. JL wrote the first draft of the manuscript. CZ contributed to the refinement of the manuscript. The final manuscript has been read and approved by all authors.

## Conflict of Interest

The authors declare that the research was conducted in the absence of any commercial or financial relationships that could be construed as a potential conflict of interest. The reviewer TH declared a shared parent affiliation with the authors CZ and LH to the handling editor at the time of review.

## Publisher's Note

All claims expressed in this article are solely those of the authors and do not necessarily represent those of their affiliated organizations, or those of the publisher, the editors and the reviewers. Any product that may be evaluated in this article, or claim that may be made by its manufacturer, is not guaranteed or endorsed by the publisher.
